# Heat stress as an innovative approach to enhance the antioxidant production in *Pseudooceanicola* and *Bacillus* isolates

**DOI:** 10.1038/s41598-020-72054-y

**Published:** 2020-09-15

**Authors:** Abdelrahim H. A. Hassan, Wael N. Hozzein, Ahmed S. M. Mousa, Walaa Rabie, Dalal Hussien M. Alkhalifah, Samy Selim, Hamada AbdElgawad

**Affiliations:** 1 Department of Food Hygiene and Control, Faculty of Veterinary Medicine, Beni-Suef University, Beni-Suef, 62511 Egypt; 2 Bioproducts Research Chair, Zoology Department, College of Science, King Saud University, Riyadh, 11451 Saudi Arabia; 3 Department of Plant Pathology, Plant Pathology Research Institute, Agricultural Research Center, Giza, Egypt; 4 Department of Biology, College of Science, Princess Nourah Bint Abdulrahman University, Riyadh, Saudi Arabia; 5 Department of Clinical Laboratory Sciences, College of Applied Medical Sciences, Jouf University, P.O. 2014, Sakaka, Saudi Arabia; 6 Botany Department, Faculty of Science, Suez Canal University, P.O. 41522, Ismailia, Egypt; 7 Botany and Microbiology Department, Faculty of Science, Beni-Suef University, Beni-Suef, 62521 Egypt

**Keywords:** Chemical biology, Microbiology, Molecular biology, Physiology, Environmental sciences

## Abstract

It is well known that the quality and quantity of bioactive metabolites in plants and microorganisms are affected by environmental factors. We applied heat stress as a promising approach to stimulate the production of antioxidants in four heat-tolerant bacterial strains (HT1 to HT4) isolated from Aushazia Lake, Qassim Region, Saudi Arabia. The phylogenetic analysis of the 16S rRNA sequences indicated that HT1, HT3 and HT4 belong to genus *Bacillus*. While HT2 is closely related to *Pseudooceanicola marinus* with 96.78% similarity. Heat stress differentially induced oxidative damage i.e., high lipid peroxidation, lipoxygenase and xanthine oxidase levels in HT strains. Subsequently, heat stress induced the levels of flavonoids and polyphenols in all strains and glutathione (GSH) in HT2. Heat stress also improved the antioxidant enzyme activities, namely, CAT, SOD and POX in all strains and thioredoxin activity in HT3 and HT4. While GSH cycle (GSH level and GPX, GR, Grx and GST activities) was only detectable and enhanced by heat stress in HT2. The hierarchical cluster analysis of the antioxidants also supported the strain-specific responses. In conclusion, heat stress is a promising approach to enhance antioxidant production in bacteria with potential applications in food quality improvement and health promotion.

## Introduction

Oxidation in biological systems and food is accountable for myriad adverse effects on human health as well as on food quality and stability. Oxidation may occur in foods during different steps of handling, leading to the formation of bad flavors, loss of essential fatty acids, fat‐soluble vitamins and other bioactive compounds, and development of potentially toxic substances, thus turning foods to unsafe for consumption^1–3^. Additionally, lipids in living organisms, particularly the polyunsaturated fatty acids in phospholipids of cell membranes, may also suffer oxidation during normal aerobic metabolism or by exposure to other oxidizing agents^4,5^. Consequently, oxidation plays a significant role in the pathogenesis of many health problems^6–8^.

Using antioxidants has become the most efficient and economical method for stabilizing food lipids and thus preventing quality deterioration of foods. As well as in medicine, antioxidants are used as health-promoting agents exploiting their ability to enhance the efficacy of the body's antioxidant defence mechanism^9^. Given the rising concern over the potential carcinogenic effects of synthetic antioxidants^10^, there is a global need to replace synthetic antioxidants with natural ones. Therefore, natural antioxidants have received substantial interest in recent years^9,11^. Particularly, antioxidants derived from small organisms such as bacteria and algae have recently been of growing interest to scientists in this field^12^.

In this vast range of different environments, living organisms are exposed to enormous changing environmental stressors including unfavorable temperature, salinity, adverse pH, high osmolarity, radiations and pollutants^13^. Heat has become one of the foremost abiotic stresses to almost all organisms, including eukaryotes and prokaryotes^14,15^, especially with the global environmental changes. Heat stress can be defined as the rise in temperature beyond a threshold level for a period of time sufficient to cause irreversible damages to organisms. In general, this transient elevation in temperature is usually 10–15 °C above ambient, however, summer heat could also reach temperature as high as 55 °C in places such as Arabian Desert. Heat stress inhibits photosynthesis in plants^16^, destroys cell membranes^17^, and induces cellular death^18^. As a result of heat stress in plants, high reactive levels of oxygen species (ROS) are generating, namely, superoxide (·O_2_^−^), hydrogen peroxide (H_2_O_2_) and hydroxyl free radical (·OH), which ultimately leads to oxidative damage^19–22^. Appropriately, many studies have addressed the response of eukaryotes to heat stress, as well as prokaryotes such as cyanobacteria, and their mechanism of tolerance to extreme temperatures^23–30^. On the contrary, to our knowledge, the number of studies that investigated the physiological and biochemical responses of bacteria to extreme heat stress is negligible.

Herein, we aimed at exploiting heat stress as a simple way to induce the antioxidant capacity and produce antioxidant metabolites from heat-tolerant bacterial isolates. Therefore, we isolated 17 bacterial isolates from Aushazia Lake at Qassim region of Saudi Arabia. Four bacterial isolates demonstrated high tolerance to high temperature (56 °C) and were named heat-tolerant strains (HT1 to HT4) throughout the study. Consequently, these 4 isolates were fully identified and sequenced. Subsequently, we investigated the mechanism triggered by these four isolates to tolerate heat stress. In detail, we explored the physiological and biochemical responses to heat stress in terms of oxidative damage markers, overall antioxidant capacity, as well as their effect on the production of antioxidant metabolites and enzymes. We demonstrated that heat stress induces the production of antioxidant metabolites and enzymes from bacteria which could be of potential application as functional additives to enhance food stability and promote human health.

## Results

### Bacterial isolates and growth capability under heat stress

Out of 17 bacterial isolates recovered from Aushazia Lake at Qassim region of Saudi Arabia, four isolates demonstrated good growth ability and colonization under heat stress (56 °C). Whereas the remaining 13 isolates could not tolerate heat stress. Consequently, these 4 heat-tolerant strains (HT1, HT2, HT3 and HT4) were selected for further identification. As well as, we investigated the physiological and biochemical responses of these HT strains to heat stress in terms of oxidative damage markers, overall antioxidant capacity, as well as their effect on the production of antioxidant metabolites and enzymes.

### Characterization and identification of HT isolates

**Figure 1:**
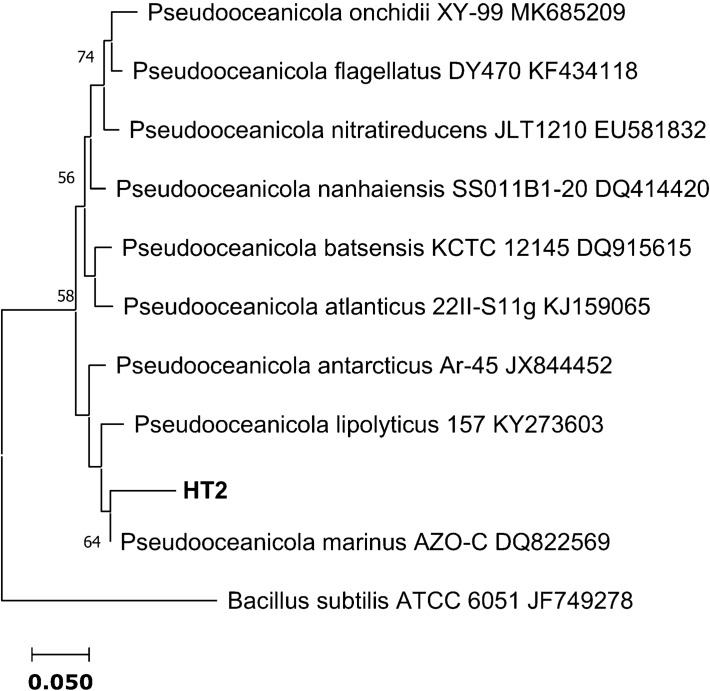
Neighbor-joining phylogenetic tree showing the relationship between the heat-tolerant isolate HT2 from the present study and the closely related species in genus *Pseudooceanicola*. *Bacillus subtilis* ATCC 6051 was used as the out‐group.

**Figure 2:**
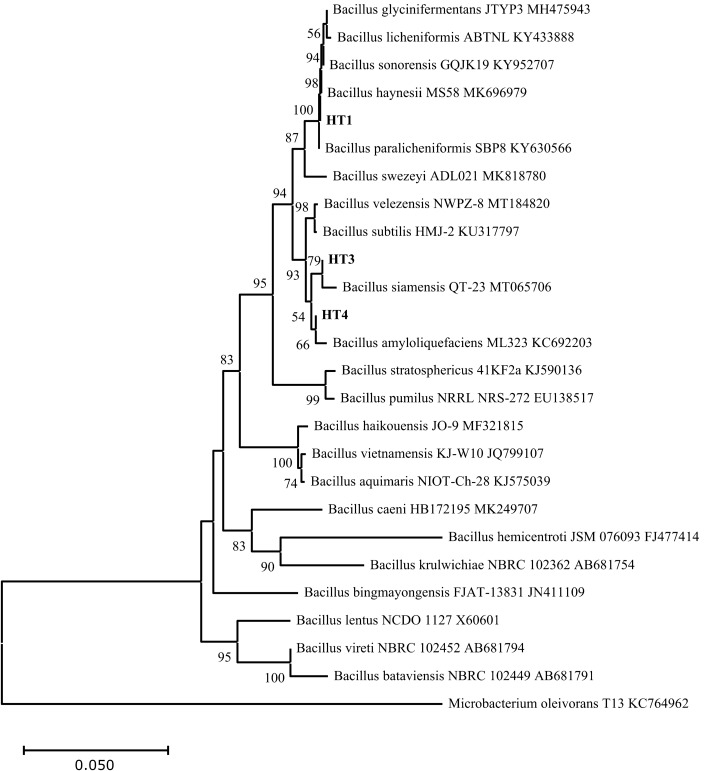
Neighbor-Joining phylogenetic tree showing the relationships between the heat-tolerant isolates (HT1, HT3 and HT4) from the present study and the closely related species of genus *Bacillus*. *Microbacterium oleivorans T13 KC764962* was used as the out‐group.

Comparison of the 16S rRNA sequences of the four HT strains with the available prokaryotic sequences in the public databases indicated that HT2 is closely related to *Pseudooceanicola marinus* with 96.78% similarity (Fig. 1). While HT1, HT3 and HT4 belong to the genus *Bacillus* spp. HT1 is closely related to *Bacillus haynesii* and *Bacillus paralicheniformis* with 99.85% similarity. Whereas, HT3 has 99.60% similarity with *Bacillus velezensis* and *Bacillus amyloliquefaciens* and HT4 has 99.45% similarity with *Bacillus amyloliquefaciens* (Fig. 2)*.*

### Oxidative damage markers under heat stress

**Figure 3:**
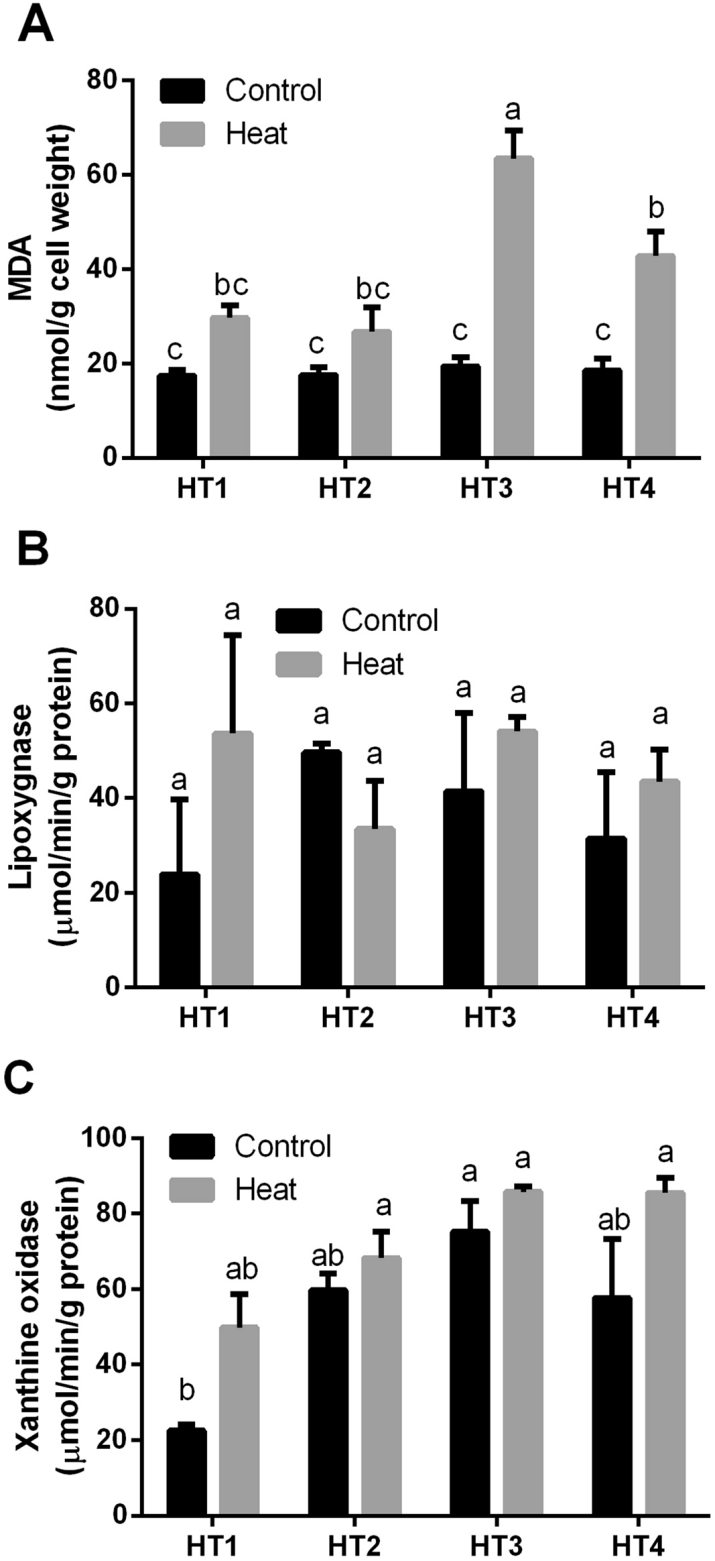
The oxidative damage markers of the four heat-tolerant bacterial isolates (HT1, HT2, HT3 and HT4) in terms of (**A**) Lipid peroxidation (MDA), (**B**) Lipoxygenase activity and (**C**) Xanthine oxidase activity under control and heat stress conditions. Data are represented by the mean of at least 3 replicates ± standard error. Different small letters (a, b, c…) above bars indicate significant differences between means at *p* < *0.05*.

Herein, the oxidative stress markers for four HT isolates which exhibited good growth capability and colonization under heat stress were determined (Fig. 3). Lipid peroxidation (MDA), lipoxygenase and xanthine oxidase of those four bacterial strains were compared under both control condition and heat stress. Under control condition, there were not any significant differences between MDA values of the four isolates at *p* < 0.05. While exposure to heat stress increased the lipid peroxidation values in all isolates, this elevation was significant in HT3 and HT4 (Fig. 3A). Regarding lipoxygenase %, heat-induced stress contributed to increasing the lipoxygenase activity % in all selected isolates except HT2, however, that elevation was not significant at *p* < 0.05 (Fig. 3B). Similarly, in xanthine oxidase %, the exposure to heat stress slightly increased its level in all selected isolates without significant difference than control (*p* > 0.05) (Fig. 3C).

### Antioxidant defense system

#### Effect of heat stress on the antioxidant capacity of HT isolates

**Figure 4:**
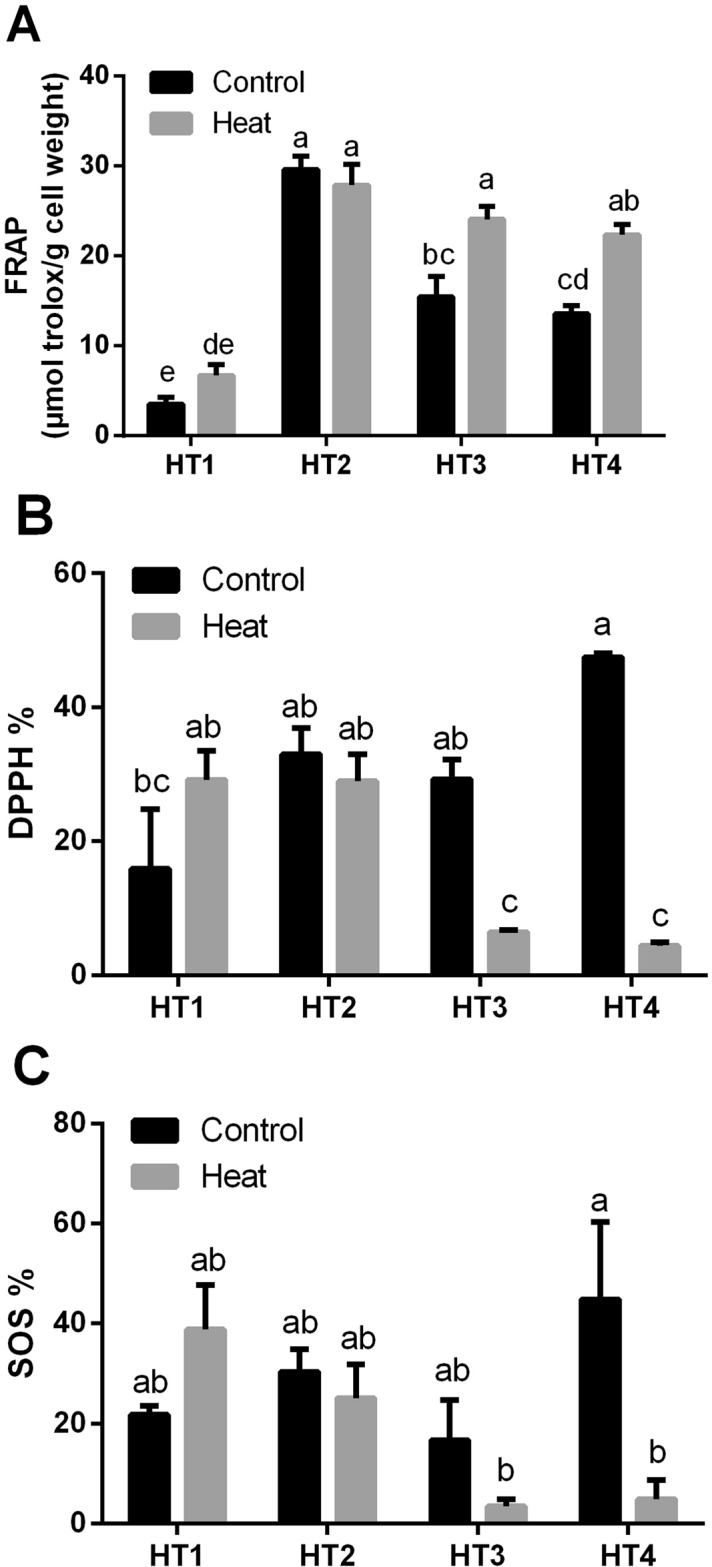
The overall antioxidant capacity of the four heat-tolerant bacterial isolates (HT1, HT2, HT3 and HT4) in terms of (**A**) FRAP, (**B**) DPPH % and (**C**) superoxide scavenging (SOS) under control and heat stress conditions. Data are represented by the mean of at least 3 replicates ± standard error. Different small letters (a, b, c…) above bars indicate significant differences between means at *p* < *0.05*.

The effect of heat-induced stress on the overall antioxidant capacity, namely, FRAP, 2,2‐diphenyl‐1‐picrylhydrazyl (DPPH) and superoxide scavenging (SOS) of the 4 selected isolates was determined (Fig. 4). The measurement of FRAP activity showed that HT1 isolate had the lowest FRAP capacity at both control and heat stress conditions, conversely, HT2 isolate showed the highest FRAP capacity at both control and heat stress conditions, without significant difference between control and stress conditions in both isolates. On the other hand, heat stress induced a significant elevation in the FRAP activity of HT3 and HT4 isolates (Fig. 4A). As regard to DPPH % activity, there were no significant differences between control and heat-stressed isolates of HT1 and HT2. On the contrary, heat stress resulted in significant reductions in the DPPH % of HT3 and HT4 isolates (*p* < 0.05) (Fig. 4B). Similarly, SOS activity of HT1 and HT2 were quite similar at both control condition and heat stress exposure. Whereas heat stress reduced SOS activity in HT3 and HT4 isolates, the reduction was significant in HT4 only (*p* < 0.05) (Fig. 4C).

#### Heat stress induced antioxidant metabolites in HT isolates

**Figure 5:**
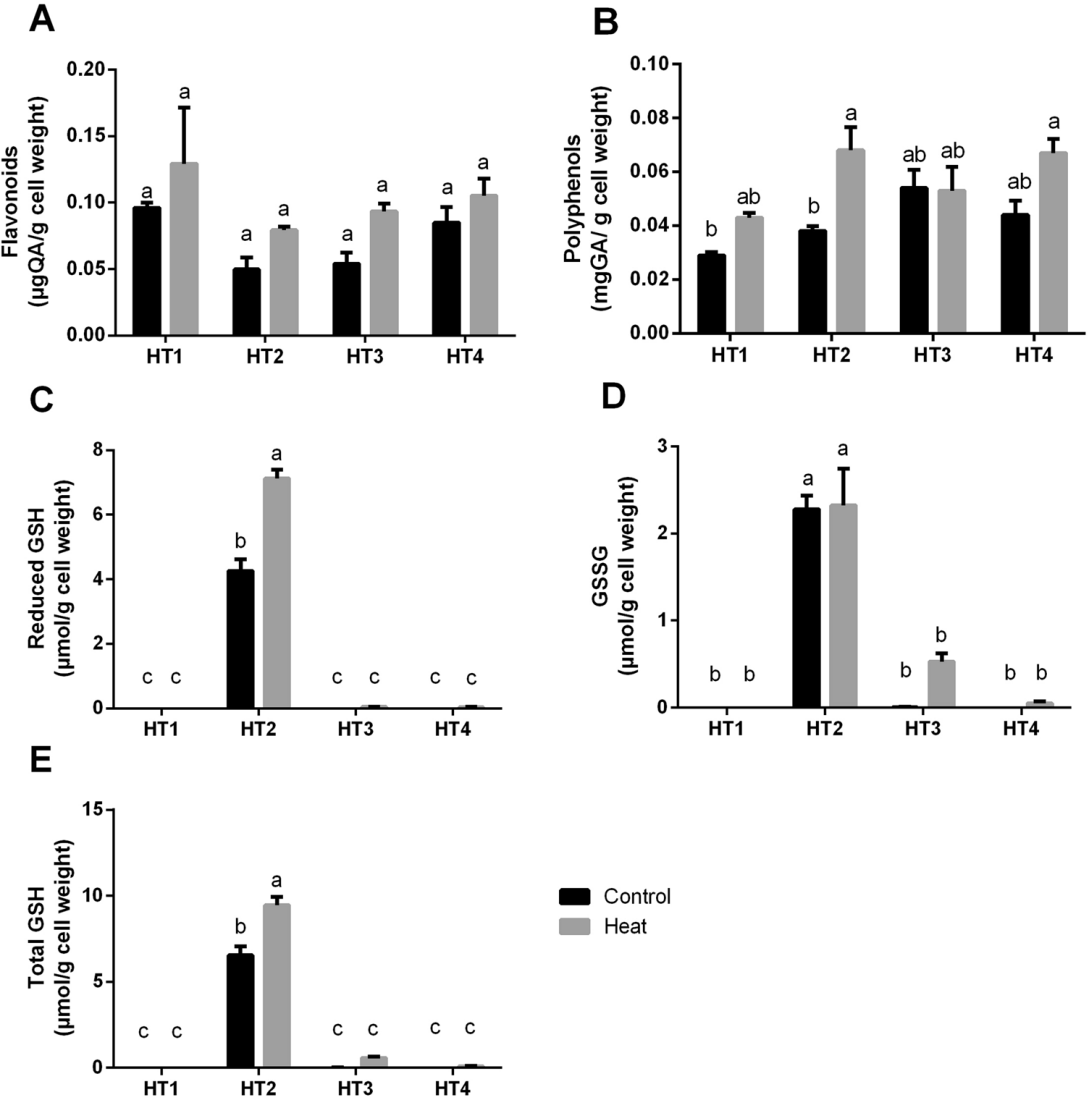
The antioxidant metabolites of the four heat-tolerant bacterial isolates (HT1, HT2, HT3 and HT4) in terms of (**A**) Flavonoids, (**B**) Polyphenols, (**C**) Reduced GSH, (**D**) oxidized GSH (GSSG) and (E) total GSH per g of bacterial cell weight under control and heat stress conditions. Data are represented by the mean of at least 3 replicates ± standard error. Different small letters (a, b, c…) above bars indicate significant differences between means at *p* < *0.05*.

To explain the changes in the total antioxidant capacity, we also measured the production rates of flavonoids and phenols by the selected isolates after heat stress exposure as compared to control condition (Fig. 5A,B). Interestingly, heat stress caused an increase in both total flavonoids and total phenols production in the four HT isolates, where this elevation was significant only in HT2 in the case of total phenols (*p* < 0.05).

Additionally, we determined the levels of GSH including the reduced GSH, oxidized GSH (GSSG) and total GSH forms (Fig. 5C–E, respectively). The results indicated that the production of all forms of GSH at both control and heat stress conditions was very negligible by all HT isolates except HT2*,* as the latter showed a considerable production of all forms of GSH, as well as, the exposure to heat stress significantly enhanced this property in this isolate in case of reduced and total GSH.

#### Heat stress induced the antioxidant enzyme activities of HT isolates

**Figure 6:**
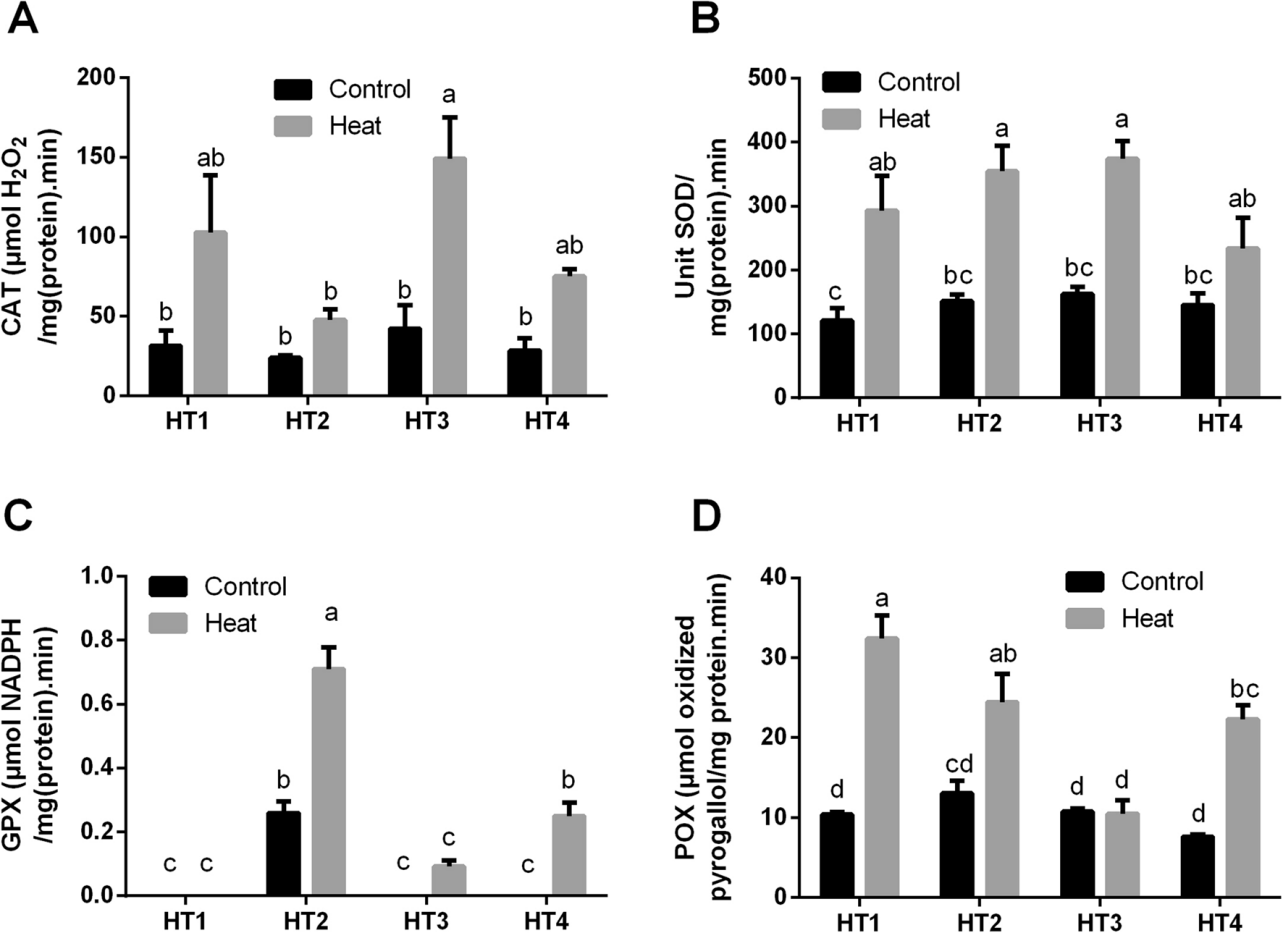
The antioxidant enzyme activities of the four heat-tolerant bacterial isolates (HT1, HT2, HT3 and HT4) including (**A**) CAT, (**B**) SOD, (**C**) GPX and (**D**) POX under control and heat stress conditions. Data are represented by the mean of at least 3 replicates ± standard error. Different small letters (a, b, c…) above bars indicate significant differences between means at *p* < *0.05*.

The production of common antioxidant enzymes that directly scavenge ROS, namely, CAT, SOD, GPX and POX were measured in the selected HT isolates under control and heat-induced stress conditions (Fig. 6). As displayed in Fig. 6A, CAT activity was quite similar in all HT isolates at control condition. While by exposing to heat stress, it was increased in the four HT isolates, this increase was significant (*p* < 0.05) in some of them. While in SOD activity in Fig. 6B, the activity at control condition was very similar in the four isolates, then heat stress improved that capacity in all isolates, which was significant in all isolates (*p* < 0.05) except in HT4. Concerning GPX activity shown in Fig. 6C, it was significantly improved by heat stress exposure in both HT2 and HT4*,* as well as, slightly improved in HT3. As it was nil or negligible in all isolates under control condition. Though, no effect was noticeable in HT1. As regard to POX activity displayed in Fig. 6D, even though, there was no significant difference between all isolates at control conditions, POX activity almost in all selected isolates was significantly improved by exposure to heat stress, only HT2 did not show significant response.

**Figure 7:**
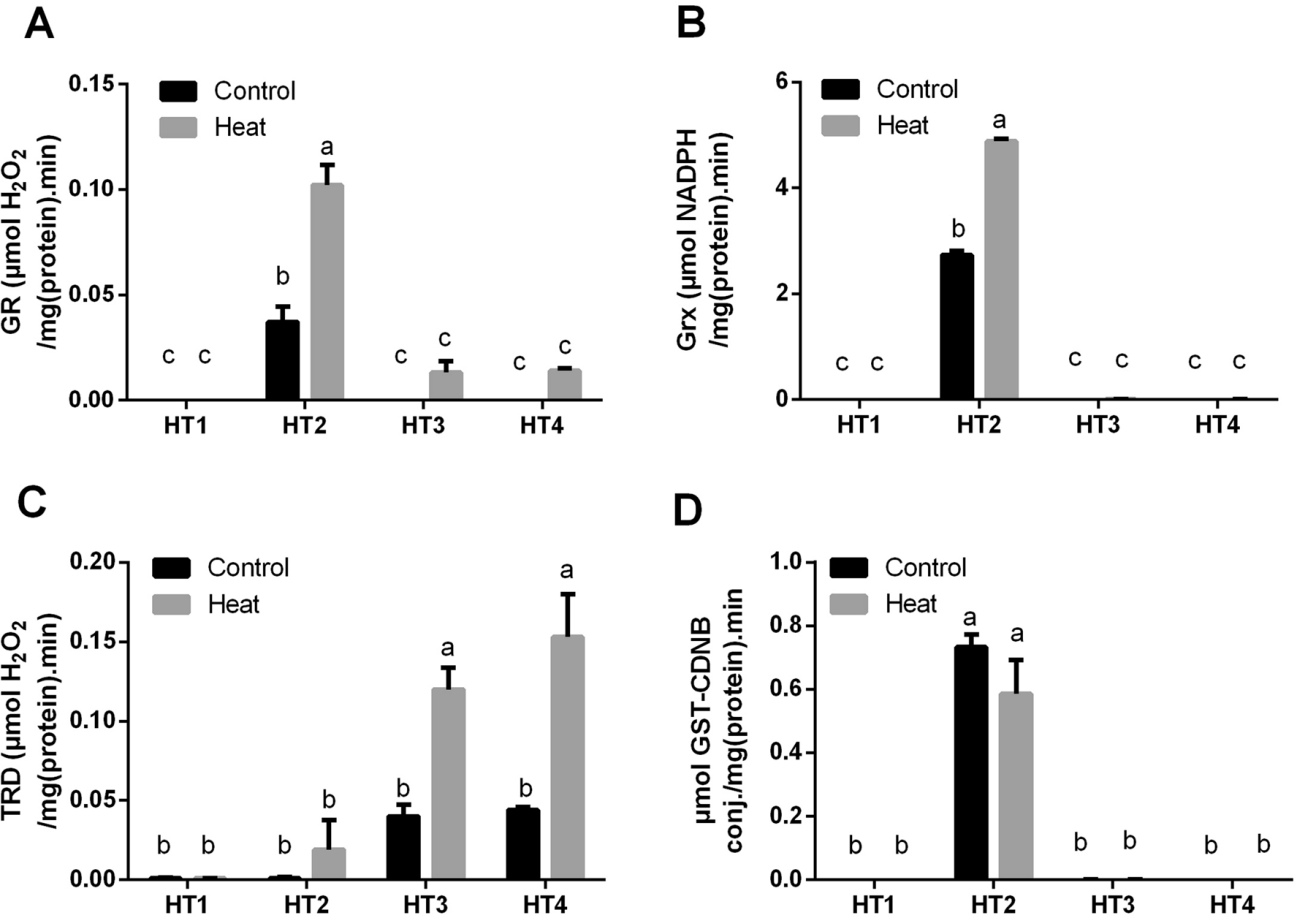
The antioxidant enzyme activities of the four heat-tolerant bacterial isolates (HT1, HT2, HT3 and HT4) including (**A**) GR, (**B**) Grx (**C**) TRD, and (**D**) GST activity under control and heat stress conditions. Data are represented by the mean of at least 3 replicates ± standard error. Different small letters (a, b, c…) above bars indicate significant differences between means at *p* < *0.05*.

Furthermore, we have explored the enzyme activities which have reducing activities to the antioxidant metabolites, i.e. GR, Grx and TRD, for the four HT isolates at control condition and heat stress (Fig. 7A–C). Apparently, HT1, HT3 and HT4 isolates had no or very negligible GR activity under control condition. Although heat stress slightly improved this activity in HT3 and HT4 isolates, it did not induce a noticeable effect on HT1. On the other side, GR activity of HT2 at control condition was significantly higher than the three other isolates (*p* < 0.05), interestingly, by heat stress exposure, this activity was significantly duplicated (*p* < 0.05) in this isolate (Fig. 7A). In regards to Grx in Fig. 7B, HT1, HT3 and HT4 did not produce Grx under control condition nor by exposure to heat stress. On the other hand, Grx activity of HT2 isolate was significantly (*p* < 0.05) improved by exposure to heat stress. While in TRD activity, HT1 and HT2 isolates did not show TRD activity at control condition nor by exposure to heat stress. Conversely, this activity in HT3 and HT4 was significantly improved (*p* < 0.05) by exposure to heat stress (Fig. 7C).

Additionally, GST activity, as an important detoxification enzyme, was quantified in the selected isolates under both control and heat stress condition (Fig. 7D). Interestingly, the obtained results of GST activity were quite similar to those of Grx in the case of HT1, HT3 and HT4 which did not show GST production under control condition nor by exposure to heat stress. While heat stress did not induce a significant effect on HT2 isolate.

#### Species-specific responses

**Figure 8:**
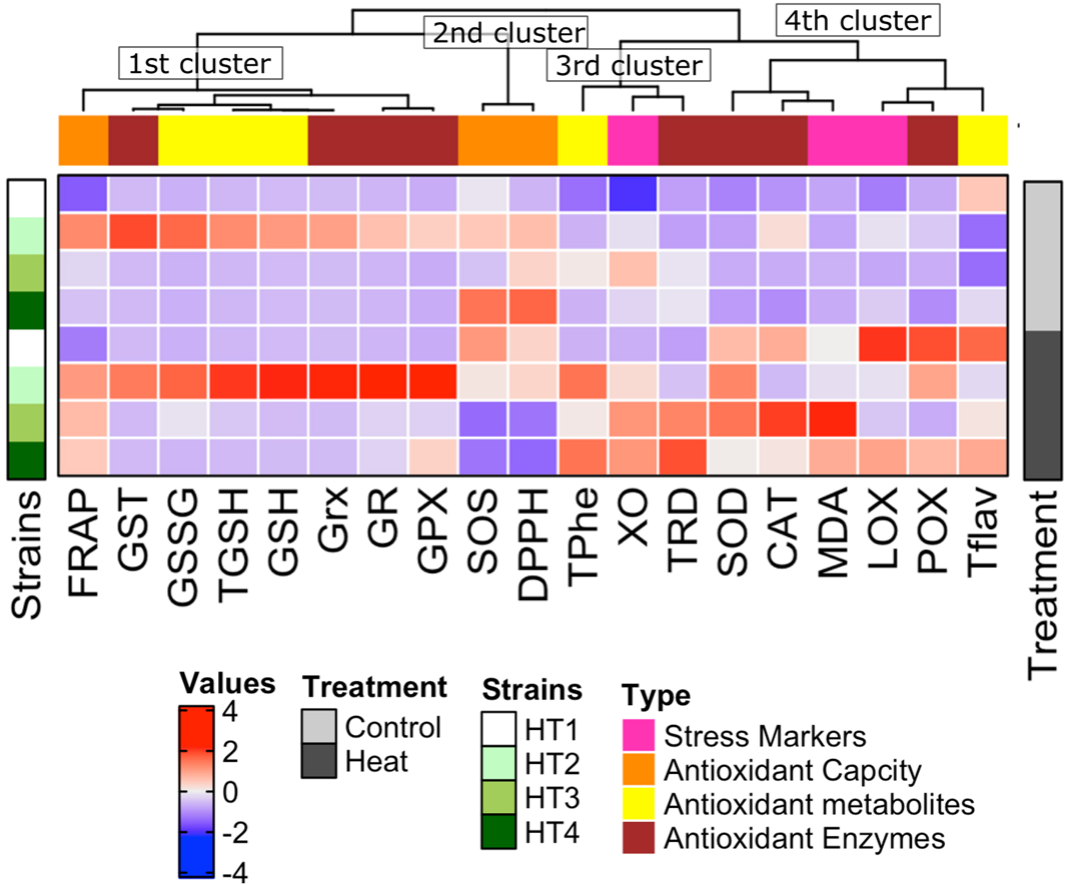
Strain-specific responses of the four heat-tolerant bacterial isolates (HT1, HT2, HT3 and HT4) to heat stress at both physiological and biochemical levels. The measured parameters represented by damage markers, overall antioxidant capacity, antioxidant metabolites and antioxidant enzymes are grouped into four main clusters based on their responses to heat stress.

The analysis of the hierarchical clustering graph (Fig. 8) suggests species-specific responses to the stress induced by heat at both physiological and biochemical levels of isolated bacterial isolates. The measured parameters represented by damage markers, overall antioxidant capacity, antioxidant metabolites and antioxidant enzymes were grouped into four main clusters based on their response to heat stress. The first cluster consisted of overall antioxidant activity (FRAP), antioxidant metabolites (reduced, oxidized and total GSH), antioxidant enzymes (GPX, GR, Grx, GST), where they were clearly high in HT2 under control condition and subsequently improved by heat stress. The second cluster was composed of overall antioxidant activities (SOS and DPPH), which were apparently high in HT2 and HT4 under control condition, as well as in HT1 under heat stress. The third cluster consisted of damage marker (xanthine oxidase), antioxidant metabolites (total phenols), antioxidant enzyme (TRD), which were apparently high in HT2, HT3 and HT4 under heat stress. Whereas the fourth cluster was composed of the damage markers (MDA and lipoxygenase), antioxidant metabolite (total flavonoids), and antioxidant enzymes (POX, CAT and SOD). They were mostly improved in all isolates by exposure to heat stress.

## Discussion

It is well known that heat stress induces multiple responses in plants and algae including morphological, physiological, biochemical, and molecular changes, as well as generation of reactive oxygen substances (ROS)^25,27,28,31^. However, to our knowledge, there is not enough data concerning the oxidative damage response of bacteria to heat stress. Oxidative stress is a consequence of exalted exposure of cells to ROS, such as superoxide anions, hydrogen peroxide, hydroxyl radicals and hydroperoxides. ROS production is an important indicator of induced-antioxidant capacity in plants or microorganisms. The production of ROS in plants, algae and microorganisms is induced by a variety of environmental stressors, such as heat, salinity, drought, high light, heavy metals and UV radiation^20,32–36^. Consequently, plant, protozoa and microbial cells use their antioxidant defense mechanisms to tackle ROS formation threats through enhancing their enzymatic and non-enzymatic antioxidant systems^32,37–40^. In this regard, a couple of studies have exploited microorganisms as a natural source of antioxidants. For instance, cyanobacteria were valuable models for studying the mechanisms behind tolerance to high salinity^35,41^. Also, Yen and Chang^42^ explored the optimum conditions for the production of antioxidants from *Aspergillus candidus*. Additionally, Rahman et al.^43^ reported that inoculating strawberry plants with *Bacillus* and *Paraburkholderia* probiotics improved the total antioxidant activities of strawberry fruits, as well as significantly increased the contents of phenolics, carotenoids, flavonoids and anthocyanins over control samples. Moreover, epiphytic marine pigmented bacteria have been recommended by Pawar et al.^44^ as a potential source of natural antioxidants.

Global warming has emerged as a serious problem affecting our ecosystem, particularly with global environmental changes. Thus, countless studies have investigated the effect of heat stress on the antioxidant capacity of higher plants, algae, and fungi^23–25,45,46^. Nevertheless, to our knowledge, the information concerning the antioxidant defense mechanisms of bacteria when exposed to heat stress is very limited. In a challenge to revolutionize promising costless approaches for enhancing the production of antioxidants from bacteria, we addressed the influence of heat stress on the overall antioxidant capacity and antioxidant production from four HT *Pseudooceanicola* and *Bacillus* strains isolated from Aushazia Lake, Qassim Region, Saudi Arabia.

The obtained results of the oxidative damage markers (MDA, lipoxygenase % and xanthine oxidase %) showed that heat stress caused varying levels of oxidative damage to the four HT isolates. Similarly in higher plants, these diverse environmental stressors affect plant processes that lead to loss of cellular homeostasis associated with the formation of ROS including H_2_O_2_^47^ and induction of oxidative damage to cellular membranes, lipids, proteins and nucleic acids^19–22^. In this context, heat stress increased lipid peroxidation (MDA) in cotton, *Gossypium hirsutum* L.^46^. Additionally, Xu et al.^30^ reported an increase in the level of MDA content in two cool-season turfgrass species with the elevation in heat stress.

In contrast to stress conditions, the oxidative damage to the four HT isolates was minimal under control conditions, which could be attributed to the low level of ROS generation under normal conditions. We noticed a correlation between the induced oxidative damage markers in stressed HT isolates with the overall antioxidant capacity in terms of DPPH, FRAP and SOS. For example, FRAP was increased in all HT isolates except HT2 (*Pseudooceanicola* strain). As control HT2 had the highest FRAP value, but exposure to heat did not induce a significant effect. Similar results were observed in DPPH and SOS in relation to each HT strain. The highest DPPH and SOS values were noticed in control HT4, which were significantly reduced by heat stress. Whereas HT1 was the only isolate that showed an increase in DPPH and SOS when exposed to heat stress. While *Pseudooceanicola* strain (HT2) demonstrated high values of DPPH and SOS and the effect of heat stress on it was negligible.

To limit oxidative damage under stress conditions, plants and/or microorganisms develop a sequence of detoxification mechanisms to control ROS level^15^. In these regards, they increase their contents of GSH, polyphenols and flavonoids to protect their cells and subcellular systems from oxidative damage^29^. So, the changes in total antioxidant capacity are most luckily explained by the changes in non-enzymatic antioxidant metabolites and enzymatic antioxidants^48^. Regarding the non-enzymatic antioxidant mechanism, all HT strains under heat stress showed elevations in flavonoids and polyphenols, where this stress-induced elevation was significant only (*p* < 0.05) in *Pseudooceanicola* strain (HT2). Similarly, Velioglu et al.^49^ attributed the elevation in the overall antioxidant activity of plants to the high contents of phenolic compounds and flavonoids. Polyphenols act as potent antioxidants due to the hydrogen-donating ability of their hydroxyl groups, and their ability to donate electrons to stop the production of free radicals. Whereas flavonoids are the greatest class of polyphenols which has scavenging or chelating actions^50^. Additionally in this study, the production of different forms of glutathione (reduced, oxidised and total glutathione) was very limited or almost nil in all HT *Bacillus* isolates, while *Pseudooceanicola* strain (HT2) showed significantly high contents of glutathione which were significantly elevated by heat stress. Interestingly, total GSH production by *Pseudooceanicola* strain was apparently much higher than that from plant sources as reported in our previous study^51^. Consequently, culturing this *Pseudooceanicola* sp. under heat stress could be a promising way for the production of glutathione that could be a good alternative to plant sources. Glutathione is a ubiquitous antioxidant tripeptide in the eukaryotic system, however, it is commonly found in Gram-negative bacteria, but is rare in Gram-positive ones^52^. In this concern, Fahey et al.^53^ surveyed the occurrence glutathione in bacteria, they detected glutathione in a few of the Gram-positive bacteria, but in most Gram-negative bacteria studied. Similar to our findings, they also added that glutathione is not produced by *Bacillus* spp. Another important finding of this study is that total GSH production by heat-stressed *Pseudooceanicola* sp. (10 µmol/g cell weight) is much higher than that of all bacterial species studied by Fahey et al. except *Escherichia coli* (27 µmol/g) and *Alcaligenes faecalis* (25 µmol/g). These results suggest heat stress as a simple costless method for the production of GSH from *Pseudooceanicola* sp., which could be of many potential applications in the food and medical industries.

Concerning the enzymatic antioxidant defense mechanism of the four HT isolates in response to heat stress, we observed that antioxidant enzymes that directly scavenge ROS, including CAT, SOD, GPX and POX were significantly enhanced in most selected HT isolates. Scavenging system includes SOD which converts the superoxide radical to H_2_O_2_, and CAT and POX which trigger the conversion of H_2_O_2_ to water and oxygen^51^. In line with our results, many studies reported that tolerance to heat stress is linked with an increase in antioxidant enzyme activities^29,32,54^. Also, Gür et al.^46^ reported increases in the activities of CAT at 45 °C, POX at 38 °C and ascorbate peroxidase (APX) at 38 °C and 45 °C in cotton (*Gossypium hirsutum* L.). Additionally, previous pieces of literature stated that the harmonized function of antioxidant enzymes such as SOD, APX, CAT and GR play an important role in the handling of ROS and regeneration of antioxidant metabolites^47,55,56^. Therefore, we also expect the same protective effect of antioxidant enzymes against ROS is applicable in bacterial isolates, similar to higher plants.

An additional factor that could contribute to counteracting the effect of oxidative stress is keeping the induced level of reduced glutathione content at the cellular level by the GR activity^24^, as well as the effect of other antioxidant-reducing enzymes including Grx and TRD and the detoxification GST enzyme. In correlation with the results of glutathione content, we noticed that GR, Grx and GST activities were detectable only in *Pseudooceanicola* isolate (HT2), and heat stress significantly enhanced these enzyme activities except GST, which was slightly reduced. While the three other isolates, HT1, HT3 and HT4 (*Bacillus* spp.) did not demonstrate any of these three enzyme activities, and the effect of heat stress was approximately absent. On the other hand, TRD activity was significantly improved in HT3 and HT4 by exposure to stress. Correspondingly, Chaitanya et al.^24^ reported an elevation in the GR activity in three heat-stressed mulberry cultivars and they attributed that to de novo synthesis.

## Conclusions

Exposing heat-tolerant bacteria to heat stress led to varying degrees of oxidative damage which were reflected by elevations in the oxidative damage markers including MDA, lipoxygenase and xanthine oxidase. Consequently, these isolates exploited their enzymatic and non-enzymatic antioxidant defense mechanisms to compensate for oxidative damage resulted from heat stress. That was mirrored by elevations in polyphenols and flavonoids, which was significant (*p* < 0.05) in *Pseudooceanicola* isolate only. Interestingly, *Pseudooceanicola* isolate produced considerable high levels of all forms of glutathione, which were significantly improved by heat stress. While all forms of glutathione were undetectable in the other isolates (*Bacillus* spp.), and heat stress effect was not found. GSH production by *Pseudooceanicola* isolate was much higher than plants and most bacterial species. Additionally, in correlation with the findings of glutathione production, GR, Grx and GST enzyme activities were noticeable only in *Pseudooceanicola* isolate (HT2), then heat stress significantly enhanced these activities. Accordingly, this study suggests heat stress as an innovative costless method to enhance the production of antioxidant metabolites and enzymes from bacteria, particularly glutathione and its related enzymes from *Pseudooceanicola* sp. These antioxidant products could be promising health-promoting postbiotics to be added to various food products and pharmaceuticals.

## Methods

### Bacterial isolation and growth under heat stress

The bacterial strains were isolated from hot marine water and sediment samples collected from Aushazia Lake at Qassim region of Saudi Arabia (26°04′08.0″N 44°09′45.1″E). The isolation was carried out on M1 medium^57^ and the selected 17 bacterial isolates were purified and preserved in sterile 20% glycerol at − 80 °C. According to meteorological data, summer temperature can exceed 50 °C at many places in Saudi Arabia including the location of our bacterial strains isolation. Thus, the bacterial isolates can be thermophile and to test the maximum ability of these isolates to survive at high temperatures, we grew them for 5 days on the same isolation medium but at different high temperature degrees; i.e. 36 (control), 46 and 56 °C. Out of 17 bacterial isolates, 4 were selected for further identification and experimentations, according to their ability to grow at the highest heat stress level (56 °C). Besides, comparing to lower temperatures; i.e. 36 and 46 °C, exposure to 56 °C for 5 days stimulated the maximum antioxidant production in the 4 selected bacterial isolates, with slight increases in H_2_O_2_ and lipid peroxidation.

### Bacterial identification and phylogenetic analysis

Four heat tolerant (HT) bacterial isolates were selected for further investigation based on their tolerance to 56 °C, while all other isolates could not survive at this high temperature. For identification of the four isolates, the genomic DNA was extracted from the bacterial biomass using the DNeasy UltraClean Microbial Kit by QIAGEN following the manufacturer instructions. PCR amplification of the 16S rRNA gene was carried out using the universal primers 27F and 1492R as described previously^58^. The sequencing of the PCR products was done by Macrogen, South Korea using standard procedures. The obtained sequences were compared with available 16S rRNA gene sequences from the DDBJ, EMBL and GenBank databases using the EzTaxon-e server^59^. Multiple alignments with sequences of the related organisms were carried out using MEGA X^60^. Phylogenetic trees were generated using the neighbor-joining method^61^. The evolutionary distances were computed using the Maximum Composite Likelihood method^62^ and are in the units of the number of base substitutions per site. The resultant tree topologies were evaluated by bootstrap analysis^63^ based on 1000 resampling.

### Determination of oxidative stress markers for HT isolates

Malondialdehyde (MDA) content, an end product of lipid peroxidation, was assayed according to Hodges et al.^64^. 10 mg of freeze-dried bacterial cells were homogenized in one mL of 80% ethanol by using MagNALyser (Roche, Vilvoorde, Belgium; 7000 rpm/1 min) and reacted with thiobarbituric acid to produce pinkish red chromogenthiobarbituric acid-malondialdehyde (TBA-MDA). Absorbance at 440, 532 and 600 nm was measured using in a microplate reader (Synergy Mx, Biotek Instruments Inc., Vermont, VT, USA). MDA content was calculated and expressed as nmol/g cell weight. Xanthine oxidase, XO (EC 1.1.3.22) was measured based on xanthine/xanthine oxidase system of O_2_^−^ generation given by Beauchamp and Fridovich^65^. XO activity was monitored by the reduction of XTT (2,3-Bis(2-methoxy-4-nitro-5-sulfophenyl)- 2H-tetrazolium-5-carboxanilide sodium salt) in the absence (blank) and presence of xanthine at 470 nm. Lipoxygenase (EC 1.13.11.12) activity was assayed spectrophotometrically at 234 nm in a Shimadzu UV-160 spectrophotometer (Shimadzu Corporation, Kyoto, Japan) according to Axelrod et al.^66^.

### Determination of the overall antioxidant capacity

Total antioxidant capacity, ferric reducing/antioxidant power assay (FRAP), was determined according to Benzie and Strain^67^ by grinding 30 mg freeze-dried bacterial cells in liquid N_2_ and extracting in 2 mL of ice-cold 80% ethanol. FRAP reagent (0.3 M acetate buffer (pH 3.6), 0.01 mM 2,4,6‒Tris(2‒pyridyl)‒s‒triazine (TPTZ) in 0.04 mM HCl and 0.02 M FeCl_3_.6H_2_O) was mixed with the extract for 30 min and measured at 600 nm using a microplate reader (Synergy Mx, Biotek Instruments Inc., Vermont, VT, USA). 6‒hydroxy‒2,5,7,8‒tetramethylchromane‒2‒carboxylic acid (Trolox) was used as standard. Also, the 1,1-diphenyl-2-picrylhydrazyl (DPPH) free radical scavenging activity was measured in 30 mg freeze-dried bacterial cells according to Cheung et al.^68^. Moreover, the superoxide scavenging activity (SOS) was estimated as described by Srinivasan et al.^69^.

### Determination of antioxidant metabolites in HT isolates

Polyphenols and flavonoids were extracted in 80% ethanol (v/v) and determined according to Zhang et al.^70^ and Chang et al.^71^, gallic acid and quercetin were used as standards, respectively. Glutathione (GSH) was determined by HPLC using the method of Casasole et al.^72^. The redox status (GSH/tGSH) was calculated as the ratio of the reduced form to the total concentration of the antioxidant^48^. All antioxidant metabolites assay analyses were performed in 5 biological replicates.

### Determination of enzymatic antioxidant activities in HT isolates

Enzyme activities were determined in a semi-high-throughput set-up^34,51^. Superoxide dismutase (SOD) activity was analysed by measuring the inhibition of nitro-blue tetrazolium (NBT) reduction (ɛ550 = 12.8 mM^–1^ cm^–1^)^73^. Peroxidase (POX) activity was determined by the oxidation of pyrogallol in 100 mM phosphate buffer (ɛ430 = 2.46 mM^–1^ cm^–1^)^74^. Catalase (CAT) activity was assayed by monitoring the H_2_O_2_ decomposition at 240 nm (ɛ240 = 39.4 M^−1^ cm^−1^)^74^. Glutathione reductase (GR) activity was measured by monitoring the decrease in NADPH (ɛ340 = 6.22 mM^–1^ cm^–1^) according to the method of Murshed et al.^75^. Its activitie was assayed in 50 mM HEPES pH 8. Glutathione *S*-transferase (GST) activity was determined by measuring conjugation of GSH to 1-chloro-2,4-dinitrobenzene (CDNB) at 340 nm^76^. Glutaredoxin (Grx) activity was determined by measuring the reduction of 2-hydroxy-ethyl-disulfide by GSH in the presence of NADPH and yeast GR^77^. Glutathione peroxidase (GPX) activity was measured as described by Drotar et al.^78^, in a coupled enzyme assay with GR, measuring the decrease in NADPH absorption. Thioredoxin (TRD) activity was determined by measuring NADPH oxidation^79^ at 340 nm.

### Statistical analysis

We carried out the statistical analysis using the SPSS statistical package (SPSS Inc., Chicago, IL, USA). One-Way Analysis of Variance (ANOVA) was done and Tukey’s Test (*p* < 0.05) was applied as the post-hoc test for separation of means. Each experiment was done with at least three replicates (n ≥ 3). Cluster analysis was performed by Pearson distance metric of the MultiExperiment Viewer (MeV)™ 4 software package (Boston, MA, USA).
